# Cancer mortality in the oldest old: a global overview

**DOI:** 10.18632/aging.103503

**Published:** 2020-09-03

**Authors:** Dana Hashim, Greta Carioli, Matteo Malvezzi, Paola Bertuccio, Samuel Waxman, Eva Negri, Carlo La Vecchia, Paolo Boffetta

**Affiliations:** 1 Tisch Cancer Institute, Icahn School of Medicine at Mount Sinai, New York, NY 10029, USA; 2 Department of Clinical Sciences and Community Health, Universitá degli Studi di Milano, Milan, Italy; 3 Department of Biomedical and Clinical Sciences, Universitá degli Studi di Milano, Milan, Italy; 4 Department of Medical and Surgical Sciences, University of Bologna, Bologna, Italy

**Keywords:** neoplasms, mortality, aged, 80 and over, centenarians

## Abstract

Background: As a higher proportion of adults live beyond 85 years, their cancer burden is expected to increase. While trends among the oldest old are established for major epithelial cancers (breast, prostate, lung, and colorectal cancers), they are less studied for minor cancers. This study describes age trends of cancer mortality, with emphasis on individuals aged 85+ years.

Results: Overall cancer mortality peaked at 85 years old and decreased or stabilized for all countries except the USA, France, and Japan, in which mortality continued to increase after age 85 years. For most countries, cancers of the oesophagus, stomach, liver, and larynx have a similar flat trend patterns across all ages. Bladder and kidney cancers as well as non-Hodgkin lymphoma, multiple myeloma, and leukemia showed a decreasing pattern after 85 years for UK, Germany, Italy and Poland. Lung cancer peaked at 80 years, although the age-specific peak among women did not follow the same pattern among all countries. Breast and prostate cancers increased after 85 years.

Conclusion: Mortality stabilized or decreased after age 85, particularly for non-hormonal cancers. Whether this reflects a true biological levelling of mortality rates, or lower validity of cancer registration among the oldest old, remains open to discussion.

Methods: Completed death data were obtained from the World Health Organization (WHO) for eight countries (2000 to 2014). Age-specific mortality rates were calculated for each 5-year age group above age 64. Joinpoint regression models were used to identify significant changes in mortality trends by age.

## INTRODUCTION

Persons aged 85 years or older comprise over 6% of all cancer cases [1, 2] and cancer is the fourth most common cause of death among the very old [3, 4]. As life expectancy lengthens [5], the number of cancer cases and cancer deaths among the elderly and the oldest old, defined as subjects older than 65 and 85, respectively, is projected to increase [6]. The global population of the oldest old is expected to more than triple between 2015 and 2050, growing from 126 to 446 million [7]. In some Asian and Latin American countries, this population is predicted to quadruple [7].

It remains unclear exactly how aging contributes to lifetime cancer risk. While mortality from other non-communicable diseases such as cardiovascular diseases, ischemic heart disease and stroke increases among the oldest old [8], the age-related increase in mortality from cancer appears to decelerate [9–11]. This evidence is paradoxical to the theory of mutation accumulation, which implies that genomic damages accumulate during one’s life course, leading to a steady increase in the risk for cancer [12, 13]. There is also the question of survivor bias, whereby individuals have who have been exposed to fewer risk factors surpass the age of individuals who were more exposed. Risk factor exposure may vary based on birth cohort, time period, and/or location. For example, exposure to a risk factors at a particular time periods may make it difficult to distinguish between whether some of the oldest old in a birth cohort were simply exposed to fewer cancer risk factors or whether the higher survival was due to their age [9]. Observed changes in mortality trends may also be an artifact of variations in diagnostic screening or testing in older age [14]. In particular, trends for less common cancers for which screening efficacy has not yet been well-established, are not yet understood among the oldest old [15].

The aim of this study is thus to determine age-specific trends of cancer mortality from 2000 to 2014 by cancer site across different countries among the oldest old.

## RESULTS

### General overview of cancer mortality

From 2000 to 2014, cancer mortality increased with each five-year age group following age 65, with the APCs between ages 65-69 and 80-84 being in the range of 25.8% (Poland) to 51.5% (Australia) among men and among women in the range of 25.5% (Poland) to 46.8% (Japan) between ages 65-69 and 85-89 for most countries (Supplementary Tables 2, 3). The overall AAPC of all cancers increased with age following 65 years old up to 95+ years old, ranging from 4.9% (Poland) to 33.6% (Australia) for men and from 11.2% (Poland) and 36.1% (Japan) for women.

**Figure 1:**
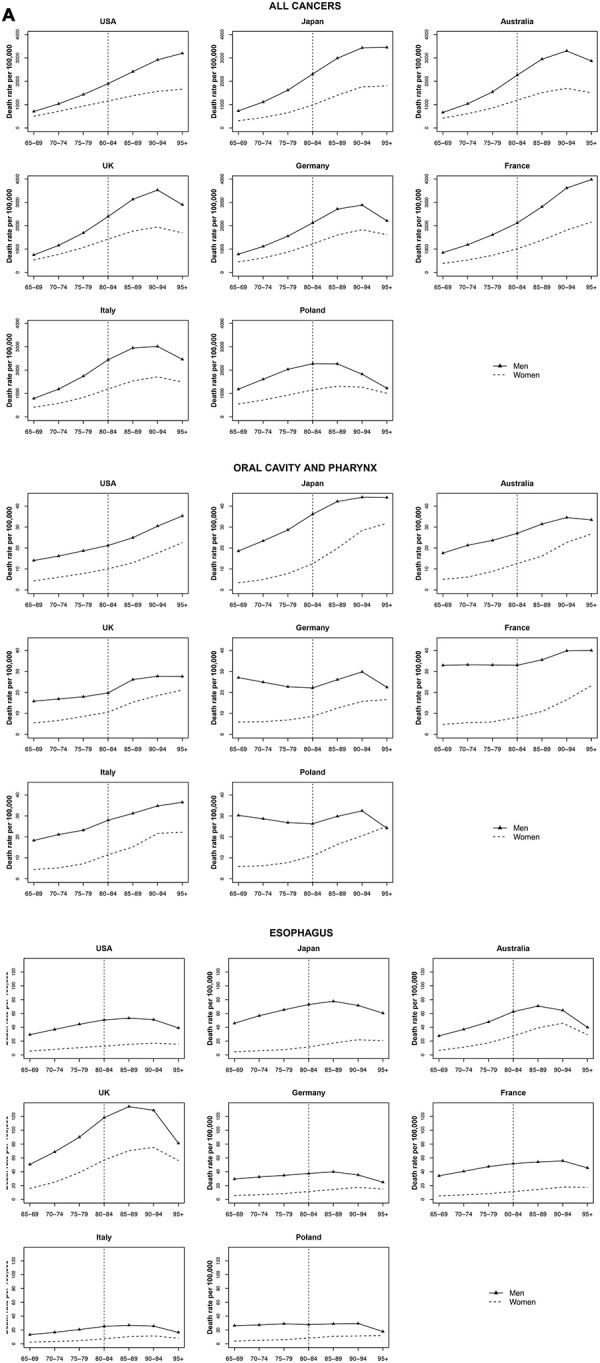
**Death rate per 100 000 persons for oral cavity and pharynx, esophagus and all cancers in men and women at age groups 65-69, 70-74, 75-79, 80-84, 85-89, 90-94, 95+ years, in selected worldwide countries.**

**Figure 2:**
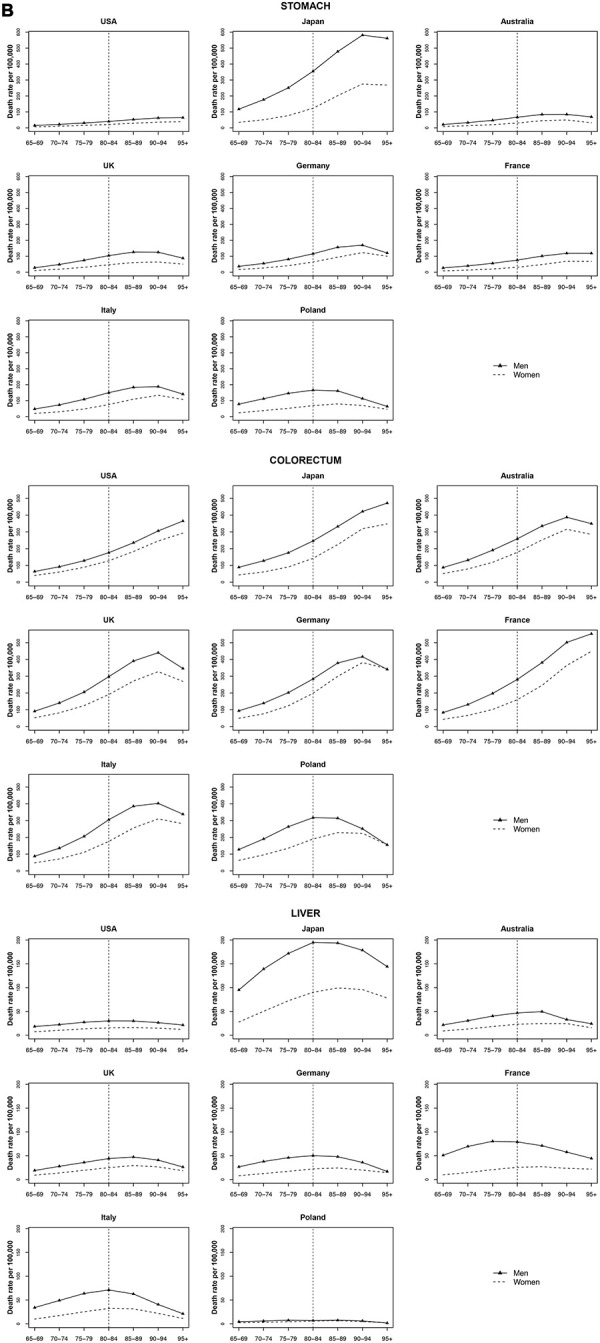
**Death rate per 100 000 persons for stomach, colorectum and liver cancers in men and women at age groups 65-69, 70-74, 75-79, 80-84, 85-89, 90-94, 95+ years, in selected worldwide countries.**

**Figure 3:**
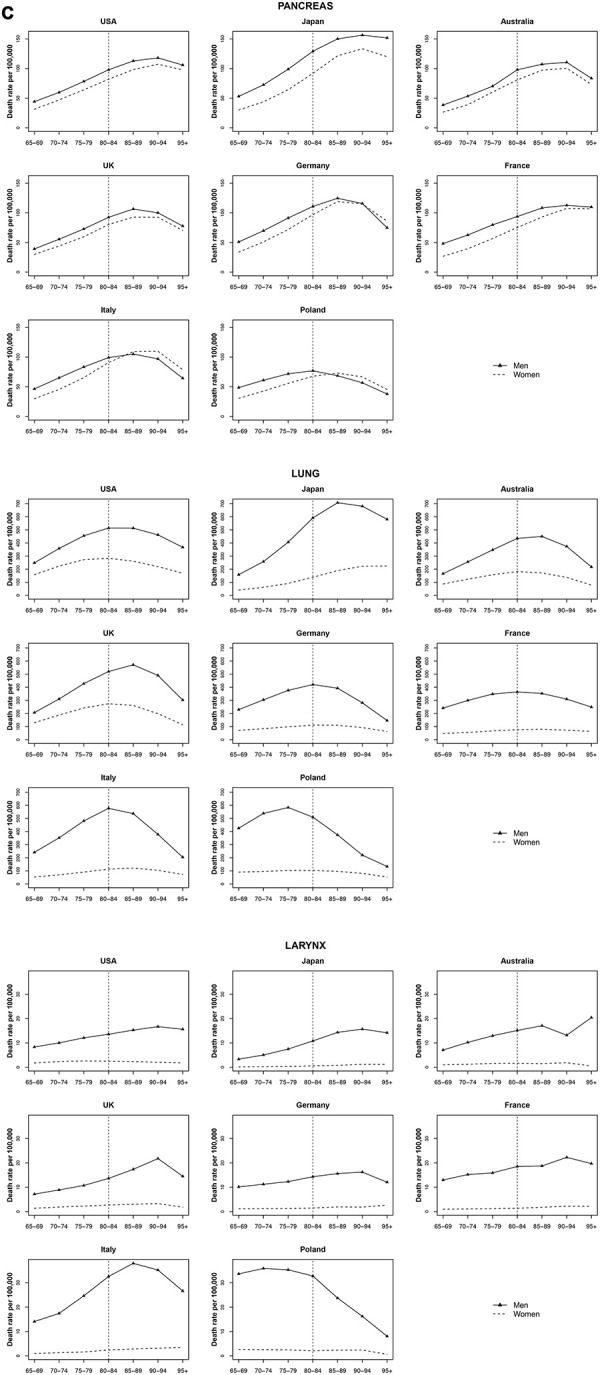
**Death rate per 100 000 persons for pancreas, larynx and lung cancers in men and women at age groups 65-69, 70-74, 75-79, 80-84, 85-89, 90-94, 95+ years, in selected worldwide countries.**

**Figure 4:**
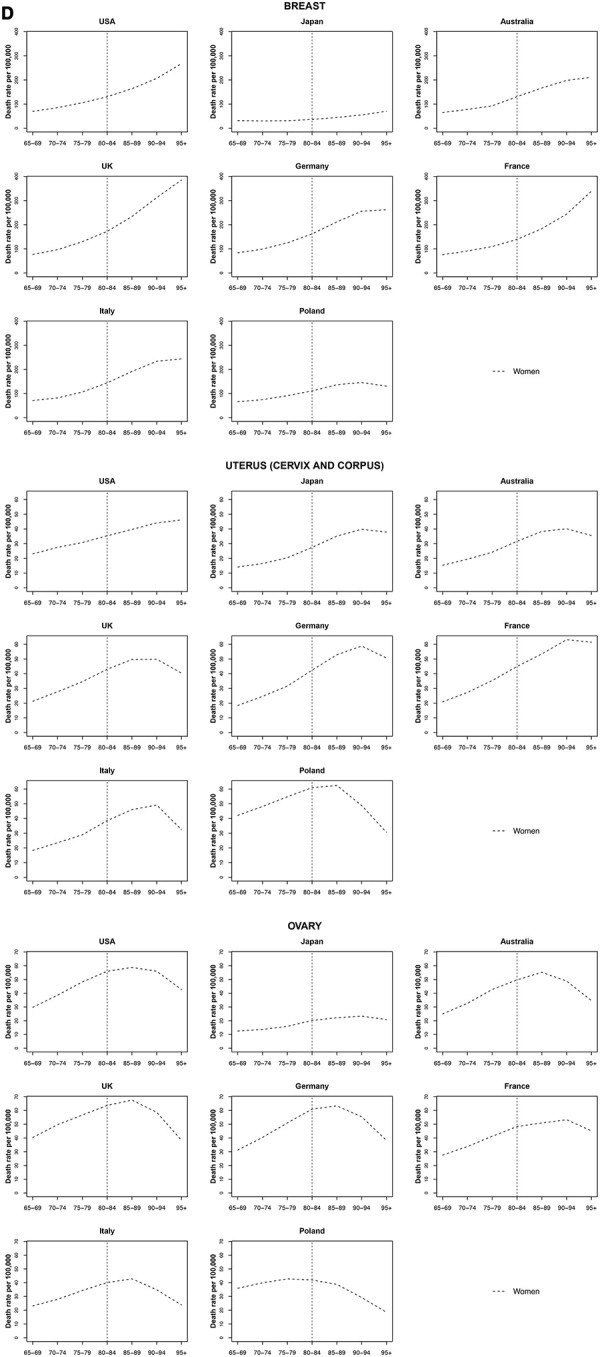
**Death rate per 100 000 persons for breast, uterus and ovary cancers in women at age groups 65-69, 70-74, 75-79, 80-84, 85-89, 90-94, 95+ years, in selected worldwide countries.**

**Figure 5:**
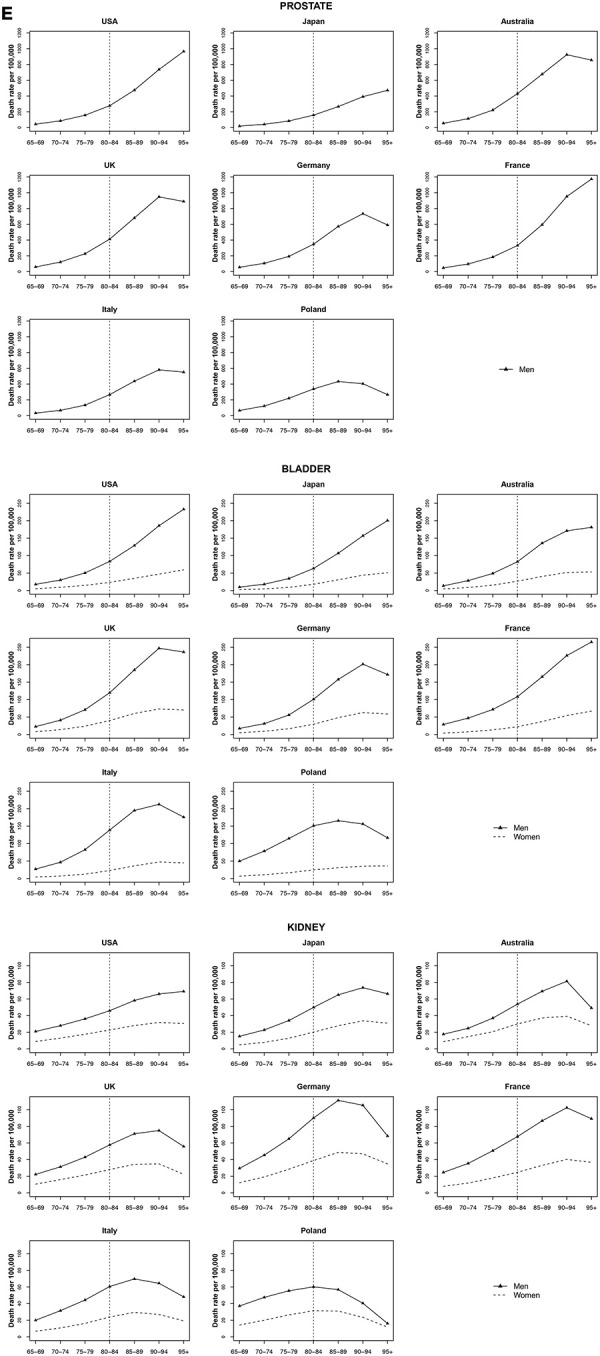
**Death rate per 100 000 persons for prostate, bladder and kidney cancers in men and women at age groups 65-69, 70-74, 75-79, 80-84, 85-89, 90-94, 95+ years, in selected worldwide countries.**

**Figure 6:**
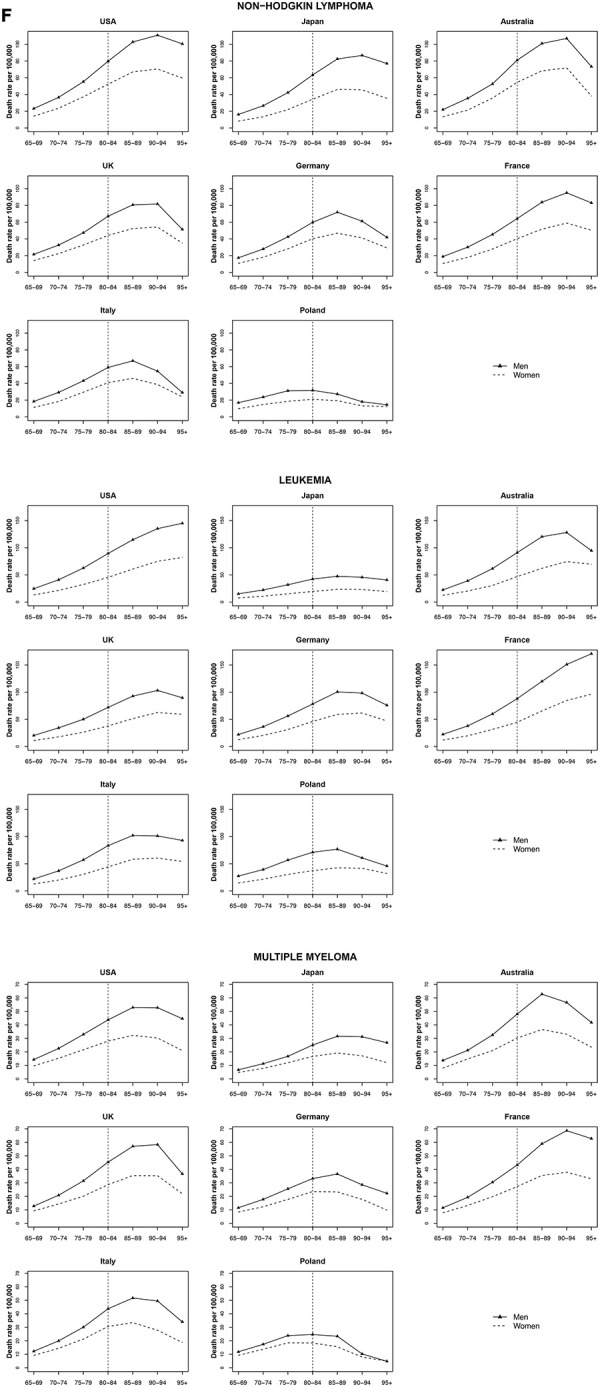
**Death rate per 100 000 persons for non-Hodgkin lymphoma, multiple myeloma and leukemia in men and women at age groups 65-69, 70-74, 75-79, 80-84, 85-89, 90-94, 95+ years, in selected worldwide countries.**

Cancer mortality in the selected countries for all cancers, for each major cancer site and both sexes are displayed in Figure 1-6. In Australia, the UK, Germany, and Italy, overall cancer mortality decreased after a peak age of 90 to 94 for both sexes. Trends in Poland started to decline earlier and showed a lower peak cancer mortality for both men (2273.45 per 100,000) and women (1314.07 per 100,000) compared to other countries. Cancer mortality continued to increase in the USA and France, and stabilized in Japan. Mortality rates are displayed in Supplementary Table 1.

### Oral cavity and pharyngeal cancers

No strong trends with age were observed among the very old for oral and pharyngeal cancers (Figure 1).

### Oesophageal cancer

A strong increase with age was not found in most countries. While a small increase was observed for some European countries, the rate change per year was not significant. Overall, the highest oesophageal cancer rates among the countries analysed were observed for the UK (peak around 134.2 per 100 000 men aged 85-89 and around 75.2 per 100 000 women aged 90 to 94) (Figure 1).

### Stomach and colorectal cancers

Stomach cancer reached a peak at age 90 to 94 and then stabilized or decelerated for both men and women in most countries. Japan had the highest increase of stomach cancer mortality with age (AAPC 31.1% for men and 49.9% for women). Colorectal cancer reached a peak at age 90 to 94 years and stabilized or decelerated in most countries, too, except for the USA, Japan, and France that continued to increase with age (Figure 2).

### Liver cancer

Liver cancer mortality rates were similar across most of countries (a peak of 60 per 100 000 or below for men and 25 per 100 000 or below for women at ages 80 to 84 years). Japan had the highest liver cancer mortality rates with a peak of 194.9 per 100 000 for men at age 80-84 and of 99.3 for women (75 per 100 000) at age 85-89 (Figure 2).

### Pancreatic cancer

For pancreatic cancer, an increase in mortality rates with age was observed for all countries among both men and women, with stable or declining trends only after 90-94 age for most countries. In the UK, Germany, and Poland, mortality rates peaked earlier at around 85 years of age in both men and women. In Italy and Poland, pancreatic cancer mortality among women overtook the mortality rate among men after age 85-89, in Germany after age 90-94 (Figure 3).

### Lung and laryngeal cancer

Among both men and women, lung cancer mortality in Australia, UK, Germany, and Italy reached a peak at age 80 to 89 (75-79 for Poland) and then decreased. At age 85-89, Japan showed the highest mortality rate of lung cancer, around 707.1 per 100 000 for men, while in women trend continued to increase with age, reaching a rate over 224.6 per 100 000 in over 95. Similar trends were found for laryngeal cancer, with however later peaks (Figure 3).

### Breast cancer

The lowest rates were observed in Japan (around 36.5 per 100 000 women aged 80 to 84). Breast cancer mortality increased monotonically with age (including Japan), except for Germany, Italy, and Poland where rates stabilized after age 90 to 94 years. The UK had the highest mortality of breast cancer after age 90 years (over 312.5 per 100 000 women). The UK also had the highest increase by quinquennial age, the mortality rate increase from 65 years old to 95+ years was 32.4% (Figure 4).

### Uterine cancer

Uterine cancer mortality seemed to decrease or stabilize at oldest old age across all countries. The decreasing trend was stronger among the oldest old in the UK, Germany, Italy and Poland. This latter had the highest mortality rates with a peak of 62.5 per 100 000 women aged 85 to 89 followed by a sharp decrease at a rate of 29.1% per year (Figure 4).

### Ovarian cancer

Ovarian cancer peaked in most countries at age 85 to 89 and then decreased. The decrease from age 85 to 89 until 95+ years was highest in Italy (-26.1%), the UK (-22.7%) and Australia (-21.4%) (Figure 4).

### Prostate cancer

Among very old men, prostate cancer mortality rates were lowest in Japan and Poland. A large increase in mortality rate was observed between ages 85 to 95+ in the USA, in Japan and France. The increase in all other countries was not significant among these age groups (Figure 5).

### Bladder cancer

Bladder cancer rates showed a strong increasing mortality in all countries with age among both men and women. For the UK, Germany, Italy, and Poland were observed decreases after age 90-94 in both sexes. In these countries, the gap in mortality rates among men and women widened after age 85 to 89 years. The exceptions among women were Japan, France, and Poland, with an increase of 29.1%, 36.8%, and 17.6% per quinquennium of age, respectively (Figure 5).

### Non-Hodgkin lymphoma, multiple myeloma, and leukemia

Mortality rate trends for non-Hodgkin lymphoma, multiple myeloma, and leukemia were similar among both men and women by age group. For Non-Hodgkin lymphoma and multiple myeloma, an increase was observed for most countries, until a peak at 85 years old, followed by a decrease. Leukemia mortality rates peaked at age 90 years in Australia and the UK; mortality rates increases occured earlier in Japan, Germany, Italy and Poland, followed by a decrease. In the USA and France, leukemia mortality rates increased for those over 90 years old (Figure 6).

## DISCUSSION

Cancers of the esophagus, stomach, colorectum, pancreas, larynx, lung, bladder, uterus, ovary, and kidney, as well as non-Hodgkin lymphoma, multiple myeloma, and leukemia showed a mortality rate acceleration with age followed by a deceleration or levelling off, particularly after 85 years. This pattern was observed for both men and women and in most countries. The acceleration-deceleration trend was especially prominent for nearly all cancers with the exception of prostate and breast cancer, which largely increased and had a later peak age of cancer mortality. Cancers of the oesophagus, stomach, liver, and larynx show a weaker relationship with age.

The relationship between age and cancer rate is consistent with cross-sectional studies that also find a rate deceleration or levelling off after a peak at 75 to 85 years old [16–20]. Cancer mortality is likely determined by factors pertaining to lifestyle and risk factor exposure, diagnosis, and treatment, that are site-specific. The mechanisms that may operate in shaping cancer mortality patterns in the elderly include (i) less intense screening, (ii) comorbidities, (iii) reduced likelihood to undergo aggressive treatment, (iv) depletion of the susceptible population, (v) disease misclassification (vi) changes in underlying risk factors (for example, hormones).

Individuals over 85 years have been mostly studied in aggregate when it comes to public health outcomes, leading to some uncertainty in rate estimations for each specific age year after 85 over shorter time periods. When pooling data by age over the course of a longer time period, however, it becomes difficult to separate period from cohort effects in the interpretation of cancer rates. The question of cancer rates among the oldest old still remains. A 2015 study using Surveillance, Epidemiology, and End Results (SEER) data which analyzed the age-period-cohort effects in individuals over 85 years in Utah [21] found that the decreasing trend in total cancer rate among the oldest old was still present, even after accounting for period and cohort effects [21]. The period effects accounted for events affecting all age groups because they occurred at one time point. For example, changes in cancer screening protocol and technology can affect all age groups to have higher or lower mortality rates, although the overall age-trend remains similar. A study of cancer trends from 1970 to 2015 [22], found an increase in prostate cancer mortality in males over age 65 years old for western countries to peak in the early 1990s, although overall, older men still had a higher mortality from prostate cancer than younger men [23, 24]. In addition to less intense screening, the effect of improved cancer treatment of aggressive prostate cancer subtypes is likely to have played a large role in decreasing mortality among the oldest old over time, although mortality rates still show an acceleration pattern and remain substantially higher than among younger men [22, 25].

Cohort effects include the unique experiences or exposures of a certain group, such as behavioral or environmental factors. Considering the variation in female bladder cancer rates among different countries, countries such as the UK and France with historically higher smoking rates in the 1950s [26] have a higher rate of bladder cancer mortality at the oldest age groups than countries with historically lower female smoking rates (for example, Italy with a 10% female smokers prevalence in the 1950s). The pattern of lung cancer mortality rates follows a similar trend with regards to tobacco smoking. The increasing lung cancer mortality among females over 85 years old in the US, for example, is preceded by increasing lung cancer rates 10 to 20 years before [27]. High mortality rates were found among men born in the 1920s [27], and who would have been in their 70s or 80s in 2000-2014, the time of this study). These cohort effects are reflected in the changes observed in mortality in particular age groups and may partly explain variations in mortality rates between countries.

Studies on the benefits of breast cancer screening often exclude women older than the age of 75 years, because screening is not recommended beyond that age [28–31]. While it has been proposed that older women may present with more aggressive or higher-risk disease [32, 33], other studies have documented that elderly women have less aggressive disease and a more favorable tumor marker profile (ER^+^ and/or PR^+^, HER2/*neu*^−^, and decreased levels of cell proliferation markers) [34, 35]. Although the exact cause is not clear, the acceleration with age for breast cancer is less steep than non-epithelial cancers [36, 37].

Existing comorbidities and whether or not a patient is able to tolerate cancer therapy can also influence cancer mortality. Patients over 85 years with colorectal cancer have more comorbidities and are treated less aggressively than their younger counterparts [38], although colorectal cancer presents more frequently among the elderly and at more advanced disease stages [39]. Advanced colorectal cancer often requires major surgery as part of treatment, and the comorbidities heavily influences surgical eligibility and potential benefit from other treatment options [40, 41]. Because patients over 85 years old are at a greater risk of non-cancer-related mortality, they frequently receive adjuvant chemotherapy and more often discontinue treatment before completion [42–45]. The combination of comorbid conditions that may cause a competing risk of death before cancer death and the avoidance of higher risk therapies that may not be beneficial, may partly explain a mortality deceleration for this age group after a peak at middle age.

There is evidence for a biological component for longer survival. Genome-wide association (GWA) studies comparing the oldest old to younger control individuals, have found multiple genes to be associated with survival and longevity [46–48]. There is also evidence for a hereditary component of age at mortality from cancer. Inherited genetic factors are estimated to play a role in about 5 to 10 percent of all cancers [49], although penetrance among families is not completely understood. Among twins, the effect of heritable factors ranged from 27 percent to 42 percent for colorectal, breast, and prostate cancer and single-gene mutations are expected to account for a fraction of these [50]. By age 70, the median cumulative risk for BRCA1 mutation carriers is about 50% to 80% for breast cancer and 24% to 40% for ovarian cancer [51]. Above age 70 to 75 years, the cumulative risk of breast cancer and ovarian cancer among BRCA1 and BRCA2 mutation carriers stabilizes [52]. Differences in breast cancer survival among BRCA2 mutation carriers are observed among patients carrying different genotype distributions. These differences may at least partly account for differences in survival among individuals with mutations of high-risk susceptibility genes. However, the frequency of mutations in known high-risk susceptibility genes as well as DNA mismatch-repair genes in hereditary nonpolyposis colorectal cancer, and the candidate gene *HPC1* in prostate cancer [50] is too low to fully explain the majority of cancer mortality cases.

This study uses estimates that reflect the quality of national mortality data based on WHO principals [55]. There is a paucity of epidemiological data regarding cancer mortality trends in the very old, and persons over 85 years are commonly excluded from clinical trials or combined in a single age group in population-based analyses [5, 53, 54]. By stratifying by country and, at the same time, pooling data by a span of 15 years, we were able to compare mortality trends among the oldest old and younger age groups. We were also able to provide an overview of trends for cancer mortality versionin total and cancer mortality by site, including less common non-epithelial cancers. As this study was limited to countries with a high standard of data quality, it is not an exhaustive view on mortality in all adults aged 85+ years worldwide. In particular, caution must be taken not to extrapolate the interpretation of these findings to low- to middle- income countries with varying access to diagnostic and treatment resources.

There is an under-ascertainment of cancer mortality at older age groups in epidemiology. Worldwide the Global Burden of Diseases have observed increases in the incidence of breast, colorectal and prostate cancer, especially in countries in transition [54]. Preventive strategies, including minimizing risk factors and optimizing screening, are likely to be critical to provide the necessary cancer care to reduce the burden of cancer in an ageing population.

## MATERIALS AND METHODS

Official death certification data from 2000 through 2014 were extracted from the World Health Organization (WHO) Statistical Information System (WHOSIS) [15] for all cancers and 17 cancer sites: oral cavity and pharynx, including lip (C00-C14), esophagus (C15), stomach (C16), colorectum (C18-C21), liver (C22), pancreas (C25), larynx (C32), lung (C33-C34), breast (C50), uterus (cervix and corpus) (C53 and C54), ovary (C56), prostate (C61), bladder (C67), kidney (C64-C65), non-Hodgkin lymphoma (C81-C90, C96), multiple myeloma (C90), leukemia (C91-C95) and total cancers (malignant and benign) excluding non-melanoma skin (C00-C96, excluding C44). We retrieved mortality figures for 8 major countries with over 20 million inhabitants and over 95% population death certification coverage [55]. This included the USA, Japan, Australia, the UK, Germany, France, Italy and Poland. Cancers were coded in the WHOSIS database, based on the 10^th^ Revision of the International Classification of Diseases (ICD) [56]. For the period 2000-2002 in Italy, the 9^th^ Revision of the ICD was used [57]. Because coding differences between various revisions were, in general minor, we recoded all data for Italy according to the 10^th^ Revision.

### Statistical analysis

Age-specific mortality rates (2000-2014) were calculated for each 5-year age group from 65 and 84, and compared to 85 to 95+ age groups. We used joinpoint regression models [58], allowing for up to two trend segments, to identify age groups for which there were significant trend changes in the age-specific mortality for the 8 countries and the 17 cancer sites under study. We computed the estimated age percent change (APC) (defined as the percent change from one 5-year age group to the next) and the average quinquennial age percent change (AAPC) over all age groups considered [59, 60].

## Supplementary Material

Supplementary Table 1

Supplementary Table 2

Supplementary Table 3

## References

[1] Pilleron S, Sarfati D, Janssen-Heijnen M, Vignat J, Ferlay J, Bray F, Soerjomataram I. Global cancer incidence in older adults, 2012 and 2035: a population-based study. Int J Cancer. 2019; 144:49–58. 10.1002/ijc.31664 29978474

[2] GLOBOCAN and World Health Organization, Department of Information, Evidence and Research, mortality database..

[3] Lahti L, Huovari J, Kainu M, Biecek P. Retrieval and analysis of Eurostat open data with the eurostat package. R Journal. 2017; 9:385–92, Version 3.3.1.3 Package URL: http://ropengov.github.io/eurostat Manuscript URL: https://journal.r-project.org/archive/2017/RJ-2017-019/index.html. 10.32614/RJ-2017-019

[4] United Nations, Department of Economic and Social Affairs, Population division. World Population Prospects, the 2015 revision..

[5] Chang AY, Skirbekk VF, Tyrovolas S, Kassebaum NJ, Dieleman JL. Measuring population ageing: an analysis of the global burden of disease study 2017. Lancet Public Health. 2019; 4:e159–67. 10.1016/S2468-2667(19)30019-2 30851869 PMC6472541

[6] Berger NA, Savvides P, Koroukian SM, Kahana EF, Deimling GT, Rose JH, Bowman KF, Miller RH. Cancer in the elderly. Trans Am Clin Climatol Assoc. 2006; 117:147–55. 18528470 PMC1500929

[7] He W, Goodkind D, Kowal PR. An aging world: 2015. Washington, DC: United States Census Bureau; 2016.

[8] Needham SL. Toward priorities for aging research. Rejuvenation Res. 2014; 17:154–56. 10.1089/rej.2013.1508 24094115 PMC3995434

[9] Salinari G. Rethinking mortality deceleration. Biodemography Soc Biol. 2018; 64:127–38. 10.1080/19485565.2018.1511414 31274349

[10] Harding C, Pompei F, Wilson R. Peak and decline in cancer incidence, mortality, and prevalence at old ages. Cancer. 2012; 118:1371–86. 10.1002/cncr.26376 21953606

[11] de Rijke JM, Schouten LJ, Hillen HF, Kiemeney LA, Coebergh JW, van den Brandt PA. Cancer in the very elderly dutch population. Cancer. 2000; 89:1121–33. 10.1002/1097-0142(20000901)89:5<1121::aid-cncr22>3.3.co;2-7 10964343

[12] Ljubuncic P, Reznick AZ. The evolutionary theories of aging revisited—a mini-review. Gerontology. 2009; 55:205–16. 10.1159/000200772 19202326

[13] Pedersen JK, Skytthe A, Christensen K. Cancer occurrence in offspring of long-lived siblings. In: Hofman A, editor. Eur J Epidemiol. Aarhus, Denmark: Springer; 2013 pp. S11–S12. EuroEpi2013.

[14] Pedersen JK, Engholm G, Skytthe A, Christensen K, and Academy of Geriatric Cancer Research (AgeCare). Cancer and aging: epidemiology and methodological challenges. Acta Oncol. 2016 (Suppl 1); 55:7–12. 10.3109/0284186X.2015.1114670 26825001 PMC4957549

[15] DeSantis CE, Miller KD, Dale W, Mohile SG, Cohen HJ, Leach CR, Goding Sauer A, Jemal A, Siegel RL. Cancer statistics for adults aged 85 years and older, 2019. CA Cancer J Clin. 2019; 69:452–67. 10.3322/caac.21577 31390062 PMC12103238

[16] Stanta G, Campagner L, Cavallieri F, Giarelli L. Cancer of the oldest old. What we have learned from autopsy studies. Clin Geriatr Med. 1997; 13:55–68. 8995100

[17] Saltzstein SL, Behling CA, Baergen RN. Features of cancer in nonagenarians and centenarians. J Am Geriatr Soc. 1998; 46:994–98. 10.1111/j.1532-5415.1998.tb02755.x 9706889

[18] Andersen SL, Terry DF, Wilcox MA, Babineau T, Malek K, Perls TT. Cancer in the oldest old. Mech Ageing Dev. 2005; 126:263–67. 10.1016/j.mad.2004.08.019 15621206

[19] Arbeev Konstantin G, Ukraintseva Svetlana V, Arbeeva Lyubov S, Yashin Anatoli I. Decline in human cancer incidence rates at old ages: Age-period-cohort considerations. Demographic Research. 2005; 12:273–300. 10.4054/DemRes.2005.12.11

[20] Harding C, Pompei F, Lee EE, Wilson R. Cancer suppression at old age. Cancer Res. 2008; 68:4465–78. 10.1158/0008-5472.CAN-07-1670 18519710

[21] Hanson HA, Smith KR, Stroup AM, Harrell CJ. An age-period-cohort analysis of cancer incidence among the oldest old, utah 1973-2002. Popul Stud (Camb). 2015; 69:7–22. 10.1080/00324728.2014.958192 25396304 PMC4428162

[22] Carioli G, Malvezzi M, Bertuccio P, Hashim D, Waxman S, Negri E, Boffetta P, La Vecchia C. Cancer mortality in the elderly in 11 countries worldwide, 1970-2015. Ann Oncol. 2019; 30:1344–55. 10.1093/annonc/mdz178 31147682

[23] U.S. Preventive Services Task Force. Screening for prostate cancer: U.S. Preventive services task force recommendation statement. Ann Intern Med. 2008; 149:185–91. 10.7326/0003-4819-149-3-200808050-00008 18678845

[24] The UK NSC recommendation on Prostate cancer screening/PSA testing in men over the age of 50. https://legacyscreening.phe.org.uk/prostatecancer.

[25] Nuhn P, De Bono JS, Fizazi K, Freedland SJ, Grilli M, Kantoff PW, Sonpavde G, Sternberg CN, Yegnasubramanian S, Antonarakis ES. Update on systemic prostate cancer therapies: management of metastatic castration-resistant prostate cancer in the era of precision oncology. Eur Urol. 2019; 75:88–99. 10.1016/j.eururo.2018.03.028 29673712

[26] Graham H. Smoking prevalence among women in the european community 1950-1990. Soc Sci Med. 1996; 43:243–54. 10.1016/0277-9536(95)00369-x 8844928

[27] Islami F, Torre LA, Jemal A. Global trends of lung cancer mortality and smoking prevalence. Transl Lung Cancer Res. 2015; 4:327–38. 10.3978/j.issn.2218-6751.2015.08.04 26380174 PMC4549470

[28] Esserman L, Shieh Y, Thompson I. Rethinking screening for breast cancer and prostate cancer. JAMA. 2009; 302:1685–92. 10.1001/jama.2009.1498 19843904

[29] Morimoto T, Endo T, Odagiri K. Role of the Quality Control Committee for Mammographic Screening: The Central Committee on Quality Control of Mammographic Screening Supported by the Japan Association of Breast Cancer Screening (in Japanese). J Jpn Assoc Breast Cancer Screen. 2000; 9:25–30. 10.3804/jjabcs.9.25

[30] Morimoto T, Okazaki M, Endo T. Current status and goals of mammographic screening for breast cancer in Japan. Breast Cancer. 2004; 11:73–81. 10.1007/BF02968007 14718797

[31] Nishide H. The guideline of quality control for screening mammography in Japan. J Med Phys. 2017 (Suppl 1); 42:29 https://inis.iaea.org/search/search.aspx?orig_q=RN:49051602

[32] Moss SM, Cuckle H, Evans A, Johns L, Waller M, Bobrow L, and Trial Management Group. of mammographic screening from age 40 years on breast cancer mortality at 10 years’ follow-up: a randomised controlled trial. Lancet. 2006; 368:2053–60. 10.1016/S0140-6736(06)69834-6 17161727

[33] Statistics and Cancer Control Division, Research Center for Cancer Prevention and Screening, National Cancer Center: about Evidence Based Cancer Screening Promotion Home Page (in Japanese). http://canscreen.ncc.go.jp/.

[34] Ravdin PM, Cronin KA, Howlader N, Berg CD, Chlebowski RT, Feuer EJ, Edwards BK, Berry DA. The decrease in breast-cancer incidence in 2003 in the united states. N Engl J Med. 2007; 356:1670–74. 10.1056/NEJMsr070105 17442911

[35] The Editorial Board of the Cancer Statistics in Japan. Cancer statistics in Japan-2007. Foundation for Promotion of Cancer Research. 2007 p. 1–110.

[36] van de Water W, Markopoulos C, van de Velde CJ, Seynaeve C, Hasenburg A, Rea D, Putter H, Nortier JW, de Craen AJ, Hille ET, Bastiaannet E, Hadji P, Westendorp RG, et al. Association between age at diagnosis and disease-specific mortality among postmenopausal women with hormone receptor-positive breast cancer. JAMA. 2012; 307:590–97. 10.1001/jama.2012.84 22318280

[37] Yancik R, Wesley MN, Ries LA, Havlik RJ, Edwards BK, Yates JW. Effect of age and comorbidity in postmenopausal breast cancer patients aged 55 years and older. JAMA. 2001; 285:885–92. 10.1001/jama.285.7.885 11180731

[38] Papamichael D, Audisio R, Horiot JC, Glimelius B, Sastre J, Mitry E, Van Cutsem E, Gosney M, Köhne CH, Aapro M, and SIOG. Treatment of the elderly colorectal cancer patient: SIOG expert recommendations. Ann Oncol. 2009; 20:5–16. 10.1093/annonc/mdn532 18922882

[39] Rim SH, Seeff L, Ahmed F, King JB, Coughlin SS. Colorectal cancer incidence in the united states, 1999-2004 : an updated analysis of data from the national program of cancer registries and the surveillance, epidemiology, and end results program. Cancer. 2009; 115:1967–76. 10.1002/cncr.24216 19235249

[40] Lemmens VE, Janssen-Heijnen ML, Houterman S, Verheij KD, Martijn H, van de Poll-Franse L, Coebergh JW. Which comorbid conditions predict complications after surgery for colorectal cancer? World J Surg. 2007; 31:192–99. 10.1007/s00268-005-0711-8 17180570

[41] Rex DK, Boland CR, Dominitz JA, Giardiello FM, Johnson DA, Kaltenbach T, Levin TR, Lieberman D, Robertson DJ. Colorectal cancer screening: recommendations for physicians and patients from the U.S. Multi-society task force on colorectal cancer. Am J Gastroenterol. 2017; 112:1016–30. 10.1038/ajg.2017.174 28555630

[42] Janssen-Heijnen ML, Maas HA, Houterman S, Lemmens VE, Rutten HJ, Coebergh JW. Comorbidity in older surgical cancer patients: influence on patient care and outcome. Eur J Cancer. 2007; 43:2179–93. 10.1016/j.ejca.2007.06.008 17681780

[43] Muss HB, Biganzoli L, Sargent DJ, Aapro M. Adjuvant therapy in the elderly: making the right decision. J Clin Oncol. 2007; 25:1870–75. 10.1200/JCO.2006.10.3457 17488985

[44] Kalsi T, Babic-Illman G, Ross PJ, Maisey NR, Hughes S, Fields P, Martin FC, Wang Y, Harari D. The impact of comprehensive geriatric assessment interventions on tolerance to chemotherapy in older people. Br J Cancer. 2015; 112:1435–44. 10.1038/bjc.2015.120 25871332 PMC4453673

[45] Edwards BK, Noone AM, Mariotto AB, Simard EP, Boscoe FP, Henley SJ, Jemal A, Cho H, Anderson RN, Kohler BA, Eheman CR, Ward EM. Annual report to the nation on the status of cancer, 1975-2010, featuring prevalence of comorbidity and impact on survival among persons with lung, colorectal, breast, or prostate cancer. Cancer. 2014; 120:1290–314. 10.1002/cncr.28509 24343171 PMC3999205

[46] Broer L, Buchman AS, Deelen J, Evans DS, Faul JD, Lunetta KL, Sebastiani P, Smith JA, Smith AV, Tanaka T, Yu L, Arnold AM, Aspelund T, et al. GWAS of longevity in CHARGE consortium confirms APOE and FOXO3 candidacy. J Gerontol A Biol Sci Med Sci. 2015; 70:110–18. 10.1093/gerona/glu166 25199915 PMC4296168

[47] Deelen J, Evans DS, Arking DE, Tesi N, Nygaard M, Liu X, Wojczynski MK, Biggs ML, van der Spek A, Atzmon G, Ware EB, Sarnowski C, Smith AV, et al. A meta-analysis of genome-wide association studies identifies multiple longevity genes. Nat Commun. 2019; 10:3669. 10.1038/s41467-019-11558-2 31413261 PMC6694136

[48] Timmers PR, Mounier N, Lall K, Fischer K, Ning Z, Feng X, Bretherick AD, Clark DW, Agbessi M, Ahsan H, Alves I, Andiappan A, Awadalla P, et al, and eQTLGen Consortium. Genomics of 1 million parent lifespans implicates novel pathways and common diseases and distinguishes survival chances. Elife. 2019; 8:e39856. 10.7554/eLife.39856 30642433 PMC6333444

[49] Fearon ER. Human cancer syndromes: clues to the origin and nature of cancer. Science. 1997; 278:1043–50. 10.1126/science.278.5340.1043 9353177

[50] Lichtenstein P, Holm NV, Verkasalo PK, Iliadou A, Kaprio J, Koskenvuo M, Pukkala E, Skytthe A, Hemminki K. Environmental and heritable factors in the causation of cancer—analyses of cohorts of twins from Sweden, Denmark, and Finland. N Engl J Med. 2000; 343:78–85. 10.1056/NEJM200007133430201 10891514

[51] Mavaddat N, Peock S, Frost D, Ellis S, Platte R, Fineberg E, Evans DG, Izatt L, Eeles RA, Adlard J, Davidson R, Eccles D, Cole T, et al, and EMBRACE. Cancer risks for BRCA1 and BRCA2 mutation carriers: results from prospective analysis of EMBRACE. J Natl Cancer Inst. 2013; 105:812–22. 10.1093/jnci/djt095 23628597

[52] Piccirillo JF, Tierney RM, Costas I, Grove L, Spitznagel EL Jr. Prognostic importance of comorbidity in a hospital-based cancer registry. JAMA. 2004; 291:2441–47. 10.1001/jama.291.20.2441 15161894

[53] Townsley CA, Selby R, Siu LL. Systematic review of barriers to the recruitment of older patients with cancer onto clinical trials. J Clin Oncol. 2005; 23:3112–24. 10.1200/JCO.2005.00.141 15860871

[54] Hutchins LF, Unger JM, Crowley JJ, Coltman CA Jr, Albain KS. Underrepresentation of patients 65 years of age or older in cancer-treatment trials. N Engl J Med. 1999; 341:2061–67. 10.1056/NEJM199912303412706 10615079

[55] World Health Organization Statistical Information System. WHO mortality database Available at: http://www.who.int/healthinfo/statistics/mortality_rawdata/en/index.html.

[56] World Health Organization. International Classification of Disease and related Health Problems: 10th revision. Geneva: World Health Organization, 1992.

[57] World Health Organization. International Classification of Disease: 9th revision. Geneva: World Health Organization, 1977.

[58] Kim HJ, Fay MP, Feuer EJ, Midthune DN. Permutation tests for joinpoint regression with applications to cancer rates. Stat Med. 2000; 19:335–51. 10.1002/(sici)1097-0258(20000215)19:3<335::aid-sim336>3.0.co;2-z 10649300

[59] Clegg LX, Hankey BF, Tiwari R, Feuer EJ, Edwards BK. Estimating average annual per cent change in trend analysis. Stat Med. 2009; 28:3670–82. 10.1002/sim.3733 19856324 PMC2843083

[60] National Cancer Institute. Joinpoint Regression Program, version 4.1.1. https://surveillance.cancer.gov/joinpoint/

