# Supplementary material for: Cancer mortality in the oldest old: a global overview
Source: Aging (Albany NY). 2020 Sep 3;12(17):16744–58. doi: 10.18632/aging.103503 (PMC7521488; doi:10.18632/aging.103503)
Supplement: Supplementary Table 1 [file aging-12-103503-s001..doc]

Supplemental Table 1. Age-specific cancer mortality rates per 100,000 from selected cancer sites at ages 69-69, 70-74, 75-79, 80-84, 85-89, 90-94 and 95+ in selected countries worldwide over the period 2000-2014.

	United States of America	Japan	France	Germany	Italy	Poland	United Kingdom	Australia	
ORAL CAVITY AND PHARYNX	Men	65-69			14.0	18.6	32.9	27.1	18.3	30.3	15.8	17.6	
		70-74			16.2	23.4	33.2	24.9	21.1	28.6	16.9	21.3	
		75-79			18.6	28.6	33.1	22.7	23.2	26.8	17.9	23.6	
		80-84			21.2	36.1	33.0	22.2	27.9	26.3	19.8	27.0	
		85-89			24.9	42.2	35.5	26.1	31.2	29.8	26.1	31.4	
		90-94			30.4	44.2	39.9	29.8	34.8	32.4	27.7	34.5	
		95+			35.3	44.1	40.0	22.5	36.5	24.1	27.6	33.4	
	Women	65-69			4.3	3.4	4.8	5.9	4.4	5.8	5.4	5.0	
		70-74			6.0	4.9	5.5	6.0	5.2	6.2	6.6	6.1	
		75-79			7.8	7.8	5.9	6.8	7.2	7.7	8.5	8.8	
		80-84			10.1	12.5	8.1	8.6	11.4	11.0	10.6	12.6	
		85-89			13.0	19.9	10.9	12.5	15.2	16.4	15.3	16.1	
		90-94			17.6	28.4	16.5	15.7	21.6	20.5	18.5	22.6	
		95+			22.6	31.8	23.2	16.6	22.2	25.0	21.1	26.7	
ESOPHAGUS	Men	65-69			29.3	45.8	34.0	29.4	13.2	26.3	50.6	27.5	
		70-74			36.8	56.7	40.6	32.4	16.6	27.3	68.6	37.0	
		75-79			44.4	65.2	47.4	34.7	20.6	29.0	90.0	47.6	
		80-84			50.6	73.0	51.8	37.4	25.1	27.8	118.2	62.6	
		85-89			53.2	77.7	54.1	39.9	26.8	28.8	134.2	70.7	
		90-94			51.1	71.6	55.7	35.3	25.6	29.3	128.9	64.5	
		95+			39.0	60.4	45.5	24.8	16.5	17.7	81.2	39.9	
	Women	65-69			5.5	4.6	5.1	5.7	2.4	3.9	15.8	6.7	
		70-74			8.0	6.0	6.4	6.6	3.5	5.0	24.8	11.2	
		75-79			10.5	7.6	8.2	8.4	4.4	6.0	38.8	17.4	
		80-84			12.7	11.5	11.2	11.3	7.2	8.2	56.8	27.3	
		85-89			15.4	17.1	14.3	14.3	10.4	10.8	70.4	39.1	
		90-94			17.0	22.0	17.8	17.4	11.4	11.3	75.2	46.0	
		95+			15.5	20.5	17.5	14.8	8.0	12.1	56.2	29.1	
STOMACH	Men	65-69			15.0	117.4	26.9	36.7	47.3	78.1	28.0	21.2	
		70-74			22.1	177.0	39.8	55.0	73.1	111.8	48.4	32.9	
		75-79			30.8	251.2	55.8	81.3	108.0	145.8	74.9	47.3	
		80-84			40.2	355.0	76.0	115.9	149.7	165.6	104.0	66.7	
		85-89			52.4	478.4	101.3	156.6	183.3	160.9	126.4	84.4	
		90-94			62.3	581.3	118.5	169.5	187.7	112.7	125.3	84.7	
		95+			64.1	561.4	118.6	120.5	140.6	63.5	88.1	68.7	
	Women	65-69			6.8	34.6	8.5	16.9	19.8	23.7	10.7	8.7	
		70-74			10.5	50.9	13.5	26.4	29.8	37.4	19.0	13.6	
		75-79			15.5	76.8	20.0	41.1	47.4	51.5	30.8	20.1	
		80-84			21.4	123.1	31.0	64.1	75.2	68.8	46.8	30.4	
		85-89			29.2	202.0	48.2	94.2	108.8	79.3	60.7	45.3	
		90-94			36.0	274.9	68.5	123.0	133.5	69.6	64.2	49.6	
		95+			39.0	268.2	67.9	100.1	107.5	45.7	50.6	32.0	
COLORECTUM	Men	65-69			63.9	89.2	83.5	93.9	87.5	127.5	91.2	87.6	
		70-74			92.1	127.8	131.2	139.1	135.4	190.3	140.6	131.7	
		75-79			128.2	175.5	197.3	202.2	205.6	263.4	205.4	191.0	
		80-84			176.0	246.0	280.8	283.8	305.1	317.5	297.3	258.8	
		85-89			235.3	332.0	381.3	378.9	385.5	314.3	390.8	335.0	
		90-94			305.9	421.6	501.6	416.5	402.6	251.2	440.2	387.3	
		95+			364.8	471.6	553.0	340.9	338.2	155.2	346.3	349.0	
	Women	65-69			40.4	43.5	43.1	49.3	48.1	63.0	52.1	51.6	
		70-74			60.5	61.2	66.1	75.9	71.1	95.4	81.7	79.0	
		75-79			89.1	90.8	101.9	123.8	110.7	135.2	124.6	118.3	
		80-84			127.5	142.2	160.0	198.4	175.5	190.2	191.1	178.2	
		85-89			183.1	224.5	245.3	299.8	256.1	228.3	271.4	252.4	
		90-94			245.8	318.9	364.0	381.7	310.0	224.1	327.4	315.6	
		95+			292.8	347.4	448.8	345.1	281.4	155.4	269.4	284.7	
LIVER	Men	65-69			18.2	95.1	50.9	26.7	34.0	4.6	18.9	21.6	
		70-74			22.3	139.1	69.6	37.9	49.1	5.9	27.7	30.5	
		75-79			27.3	171.8	80.4	45.9	63.8	7.5	35.9	40.2	
		80-84			30.1	194.9	79.0	50.2	71.1	6.7	44.1	46.8	
		85-89			29.9	193.8	71.0	47.9	62.7	7.5	47.3	49.5	
		90-94			26.4	178.6	57.7	35.9	40.6	6.2	40.8	32.9	
		95+			21.3	144.2	44.3	16.9	21.2	1.6	26.3	24.1	
	Women	65-69			7.4	27.8	9.9	8.0	10.0	2.2	9.2	8.9	
		70-74			10.2	50.1	14.7	12.5	17.4	3.1	13.7	13.0	
		75-79			13.4	72.4	20.7	17.2	25.4	4.3	19.5	18.2	
		80-84			15.3	90.3	25.6	22.3	32.2	5.8	24.9	23.0	
		85-89			16.2	99.3	26.9	24.2	31.5	6.5	29.4	24.4	
		90-94			14.6	95.9	23.4	19.8	21.9	4.8	26.6	24.4	
		95+			12.1	78.3	21.8	14.6	11.3	2.2	18.4	15.6	
PANCREAS	Men	65-69			44.2	53.1	48.1	50.9	46.3	48.6	39.1	38.5	
		70-74			59.8	72.5	62.5	69.7	65.0	61.0	55.5	53.5	
		75-79			78.3	98.9	79.7	91.3	83.4	71.8	72.8	70.2	
		80-84			97.9	129.2	93.7	110.8	99.2	76.9	92.5	97.9	
		85-89			112.8	150.2	108.3	124.6	105.2	68.6	106.4	107.3	
		90-94			118.0	156.4	112.6	115.7	97.0	56.6	100.0	110.5	
		95+			105.9	151.7	109.8	74.9	64.4	37.8	77.7	83.5	
	Women	65-69			31.4	30.0	26.8	33.9	29.8	30.5	29.8	26.5	
		70-74			47.1	43.9	39.4	50.9	45.5	42.7	44.1	39.0	
		75-79			64.2	64.4	56.6	71.9	65.4	55.7	59.7	60.3	
		80-84			81.9	91.9	75.1	97.0	90.8	67.7	80.4	80.9	
		85-89			98.2	121.6	93.0	119.0	109.3	73.1	92.7	97.2	
		90-94			107.4	133.4	107.5	115.1	109.8	66.6	92.4	100.6	
		95+			97.8	120.1	106.9	85.9	78.9	45.4	70.5	73.2	
LARYNX	Men	65-69			8.4	3.3	13.0	10.1	14.1	33.6	7.2	7.1	
		70-74			10.0	5.1	15.2	11.2	17.4	35.9	8.9	10.2	
		75-79			12.1	7.5	15.9	12.3	24.6	35.3	10.7	12.9	
		80-84			13.6	10.8	18.5	14.3	32.6	32.8	13.6	15.2	
		85-89			15.3	14.4	18.7	15.6	38.0	23.7	17.4	17.1	
		90-94			16.7	15.7	22.3	16.2	35.2	16.2	21.8	13.2	
		95+			15.6	14.2	19.7	12.1	26.6	8.0	14.5	20.4	
	Women	65-69			1.8	0.2	1.0	1.1	1.0	2.6	1.3	1.0	
		70-74			2.4	0.2	1.1	1.2	1.3	2.5	1.9	1.3	
		75-79			2.6	0.3	1.2	1.2	1.6	2.4	2.3	1.6	
		80-84			2.5	0.6	1.3	1.5	2.4	2.0	2.7	1.7	
		85-89			2.4	0.8	1.8	1.9	2.8	2.3	3.0	1.5	
		90-94			2.1	1.2	2.2	1.8	3.1	2.4	3.3	1.9	
		95+			1.8	1.2	2.2	2.7	3.5	0.6	1.8	0.6	
LUNG	Men	65-69			248.4	158.5	241.3	229.4	241.1	425.2	206.7	166.0	
		70-74			358.2	257.9	299.5	304.0	352.1	538.2	310.4	256.0	
		75-79			455.0	406.1	348.2	376.3	480.8	583.3	427.6	347.2	
		80-84			514.1	591.5	364.5	420.8	577.2	509.9	520.1	435.1	
		85-89			513.2	707.1	352.6	392.8	536.6	374.3	570.8	449.8	
		90-94			461.9	680.6	309.7	281.2	377.8	220.0	490.1	373.9	
		95+			367.4	580.7	248.9	146.5	204.2	133.4	302.6	217.2	
	Women	65-69			157.9	41.5	47.7	71.2	53.4	90.5	129.7	87.5	
		70-74			223.8	61.7	55.4	84.0	69.8	95.6	187.7	123.9	
		75-79			272.8	91.5	67.2	98.1	90.7	102.7	242.1	157.7	
		80-84			283.5	138.9	76.3	111.6	113.3	102.8	274.2	181.9	
		85-89			261.3	189.4	79.4	110.4	121.3	96.2	261.3	171.6	
		90-94			219.7	223.0	72.7	92.3	104.4	81.2	198.6	137.4	
		95+			169.8	224.6	63.3	63.1	72.5	52.5	114.6	79.0	
BREAST	Women	65-69			70.0	31.8	76.0	83.6	71.4	66.2	76.8	65.4	
		70-74			85.3	30.2	91.1	99.2	82.2	75.0	96.7	77.8	
		75-79			105.6	31.2	109.7	124.7	106.5	91.4	129.6	92.7	
		80-84			130.3	36.5	139.3	162.2	144.3	111.2	172.3	130.7	
		85-89			163.8	44.5	183.8	211.9	191.8	136.8	233.9	166.8	
		90-94			206.5	55.3	243.8	255.8	234.0	146.2	312.5	198.2	
		95+			267.7	70.4	338.9	262.1	243.8	131.0	385.3	210.9	
UTERUS (CERVIX AND CORPUS)	Women	65-69			23.1	14.2	20.8	18.4	18.3	42.1	21.3	15.4	
		70-74			27.5	16.5	27.3	24.6	23.5	48.1	27.7	19.4	
		75-79			30.8	20.4	35.4	31.6	28.9	54.8	34.6	24.2	
		80-84			35.4	27.5	44.9	42.4	38.6	61.0	43.0	31.6	
		85-89			39.7	35.2	53.5	52.9	46.0	62.5	49.7	38.3	
		90-94			44.1	39.8	63.1	58.8	49.1	48.8	49.9	40.1	
		95+			46.1	37.9	61.4	50.7	32.4	30.6	40.4	35.5	
OVARY	Women	65-69			29.7	12.5	27.6	31.2	23.2	36.0	40.4	25.0	
		70-74			38.6	13.7	33.8	40.4	27.9	39.9	49.6	32.6	
		75-79			48.2	15.9	41.2	50.9	34.3	42.8	56.7	42.8	
		80-84			55.9	20.2	48.3	61.0	40.2	42.0	63.7	49.7	
		85-89			58.8	22.2	50.9	63.3	42.8	38.8	67.5	55.2	
		90-94			56.0	23.3	53.3	55.4	34.8	29.2	58.7	48.8	
		95+			42.7	20.9	45.2	38.2	23.8	18.5	38.2	34.7	
PROSTATE	Men	65-69			44.1	19.2	47.1	55.6	32.8	65.2	59.1	54.9	
		70-74			85.4	41.8	96.4	105.2	66.8	122.0	121.2	113.1	
		75-79			156.9	83.8	184.6	192.9	133.0	220.1	226.4	221.7	
		80-84			277.4	158.3	330.6	348.2	266.4	338.7	411.5	430.5	
		85-89			474.9	266.2	594.6	572.3	436.9	432.7	680.8	676.9	
		90-94			736.5	391.9	952.1	734.4	581.0	404.5	947.7	924.2	
		95+			966.1	472.6	1173.4	590.8	551.3	266.1	889.6	855.8	
BLADDER	Men	65-69			17.8	9.9	28.8	17.5	27.0	49.8	22.7	13.5	
		70-74			30.0	18.0	46.9	31.0	46.4	78.4	41.0	28.2	
		75-79			50.3	34.3	71.7	55.9	82.5	114.6	70.9	48.9	
		80-84			83.5	63.3	108.3	100.9	138.7	151.1	119.6	82.5	
		85-89			128.8	106.7	165.4	157.7	194.8	165.5	185.0	135.7	
		90-94			185.7	156.7	225.9	201.6	212.4	156.2	247.1	171.0	
		95+			232.8	200.4	265.0	171.7	175.9	116.6	236.4	181.0	
	Women	65-69			5.2	2.4	4.0	5.2	4.3	6.7	8.0	4.5	
		70-74			9.1	4.6	7.6	9.2	7.1	10.8	14.0	8.6	
		75-79			14.8	9.0	13.2	16.6	12.4	16.5	23.9	15.3	
		80-84			23.2	17.9	22.1	29.4	22.9	24.6	39.8	26.8	
		85-89			35.0	30.7	37.0	48.3	36.2	30.9	60.3	40.3	
		90-94			46.9	43.9	54.4	62.8	47.5	35.3	73.1	51.8	
		95+			59.4	50.9	67.0	58.6	44.8	36.2	70.2	53.2	
KIDNEY	Men	65-69			21.0	15.1	24.7	29.6	20.0	37.1	22.3	17.6	
		70-74			27.9	22.8	35.4	45.4	31.4	47.5	31.3	24.7	
		75-79			36.1	34.1	50.7	65.0	44.3	55.4	42.9	37.0	
		80-84			45.9	49.9	67.7	90.1	60.5	60.2	57.7	53.9	
		85-89			58.2	65.0	86.6	111.2	69.7	56.8	71.1	69.3	
		90-94			66.0	73.6	102.4	105.4	64.5	40.4	74.9	81.3	
		95+			69.1	66.3	89.1	68.4	48.1	16.1	55.8	49.2	
	Women	65-69			8.9	4.9	7.9	12.2	6.8	14.2	10.5	8.6	
		70-74			12.9	7.8	11.8	19.1	10.7	20.0	15.9	14.8	
		75-79			17.6	12.8	17.9	28.5	16.2	26.4	21.5	20.6	
		80-84			22.7	20.2	24.8	38.9	23.9	31.4	28.1	30.0	
		85-89			28.0	27.8	33.1	48.7	29.4	31.0	34.3	37.1	
		90-94			31.8	33.7	40.2	46.9	26.9	23.5	34.9	39.3	
		95+			30.5	31.0	36.7	34.8	19.3	11.7	22.2	27.9	
NON-HODGKIN LYMPHOMA	Men	65-69			23.2	16.1	19.0	17.4	18.4	17.0	21.6	22.0	
		70-74			36.5	26.6	30.2	28.0	29.2	23.7	32.7	35.4	
		75-79			55.3	42.2	45.3	42.5	43.1	31.1	47.3	52.6	
		80-84			79.7	63.3	64.3	59.8	59.0	31.7	67.1	81.0	
		85-89			102.8	82.5	83.9	71.9	66.9	27.2	80.8	101.0	
		90-94			110.8	86.7	95.1	61.2	54.6	18.1	81.8	107.0	
		95+			100.5	77.0	83.1	42.0	29.1	14.5	51.4	73.3	
	Women	65-69			14.1	8.2	10.6	10.8	11.3	9.8	14.2	13.4	
		70-74			23.6	13.4	18.0	18.1	18.4	14.7	22.5	21.4	
		75-79			37.2	21.9	27.9	28.2	29.5	18.6	32.7	35.7	
		80-84			52.3	34.3	40.2	40.2	40.8	21.0	44.3	54.4	
		85-89			67.0	46.0	51.7	46.8	46.0	19.3	52.4	68.3	
		90-94			70.5	45.6	59.0	41.3	38.6	13.1	54.2	71.8	
		95+			59.7	35.5	50.4	29.3	23.8	12.4	35.0	38.2	
MULTIPLE MYELOMA	Men	65-69			14.3	6.7	11.5	11.6	12.3	11.8	12.8	13.7	
		70-74			22.6	11.3	19.3	17.7	19.9	17.4	20.8	21.2	
		75-79			32.9	16.7	30.4	25.6	30.0	23.8	31.5	32.6	
		80-84			43.7	25.1	43.2	33.2	43.7	24.7	45.3	48.0	
		85-89			52.9	31.6	58.9	36.5	51.7	23.3	57.1	62.8	
		90-94			52.7	31.2	68.7	28.6	49.5	10.3	58.3	56.6	
		95+			44.6	26.9	62.8	22.3	34.0	4.8	36.7	41.8	
	Women	65-69			9.7	4.8	7.8	8.4	9.2	9.3	9.2	8.0	
		70-74			15.3	7.8	13.3	12.3	14.4	13.7	14.3	14.8	
		75-79			21.6	12.0	19.7	17.7	21.3	18.5	20.1	20.9	
		80-84			28.0	16.6	27.3	23.5	30.6	18.4	28.6	30.2	
		85-89			32.3	19.2	35.4	23.3	33.5	15.6	35.4	36.6	
		90-94			30.3	17.1	37.9	17.8	27.8	8.0	35.3	33.1	
		95+			21.0	12.1	33.0	9.9	18.7	4.9	22.0	23.5	
LEUKEMIA	Men	65-69			24.7	15.2	22.4	22.1	22.0	27.4	20.3	22.5	
		70-74			41.0	22.3	37.9	36.4	36.9	39.3	34.3	39.1	
		75-79			62.7	31.9	59.9	56.1	57.1	56.7	50.2	61.6	
		80-84			89.4	42.4	88.1	78.3	83.3	71.1	71.9	91.0	
		85-89			114.8	47.5	119.8	100.6	102.1	76.8	92.8	120.3	
		90-94			135.3	45.7	151.0	98.3	101.2	60.7	103.2	128.1	
		95+			145.1	40.8	170.7	76.0	93.0	45.8	89.7	94.7	
	Women	65-69			13.2	7.6	11.8	12.6	12.8	14.6	11.0	12.5	
		70-74			21.5	10.8	19.7	20.4	20.0	21.5	17.8	20.4	
		75-79			32.4	15.3	31.4	31.2	30.4	30.4	26.3	30.5	
		80-84			45.5	19.6	44.5	46.1	44.2	37.2	37.8	47.0	
		85-89			60.9	23.5	65.5	59.1	58.2	42.6	51.4	62.2	
		90-94			75.1	23.2	84.8	61.6	60.4	41.6	62.5	74.6	
		95+			82.2	19.4	96.2	47.2	54.1	32.1	59.3	69.6	
ALL CANCERS	Men	65-69			706.6	731.0	846.7	778.7	786.6	1184.4	748.9	665.9	
		70-74			1031.9	1110.0	1184.4	1111.3	1185.9	1608.2	1156.2	1036.4	
		75-79			1430.8	1617.7	1609.9	1553.0	1738.7	2035.2	1690.5	1543.4	
		80-84			1897.6	2309.3	2114.2	2119.3	2441.7	2273.5	2391.5	2262.2	
		85-89			2410.7	2985.3	2809.5	2712.1	2947.3	2267.6	3127.2	2944.7	
		90-94			2916.2	3430.8	3612.6	2882.2	3014.5	1825.5	3526.1	3298.5	
		95+			3196.7	3448.3	3975.0	2209.5	2462.2	1226.7	2899.7	2867.2	
	Women	65-69			503.9	311.0	383.8	449.1	412.0	551.1	534.7	420.6	
		70-74			711.3	446.0	528.6	617.5	575.5	719.0	770.8	610.6	
		75-79			937.0	653.3	730.0	868.4	825.6	929.7	1066.6	852.2	
		80-84			1155.4	978.8	1008.7	1217.2	1189.8	1150.9	1425.7	1188.9	
		85-89			1381.3	1402.4	1376.5	1602.9	1540.2	1314.1	1770.2	1514.6	
		90-94			1569.2	1760.9	1812.8	1827.4	1711.9	1266.7	1937.9	1698.8	
		95+			1657.9	1804.5	2155.7	1614.5	1491.0	1009.1	1694.6	1507.7	
