# Supplementary material for: Cancer mortality in the oldest old: a global overview
Source: Aging (Albany NY). 2020 Sep 3;12(17):16744–58. doi: 10.18632/aging.103503 (PMC7521488; doi:10.18632/aging.103503)
Supplement: Supplementary Table 2 [file aging-12-103503-s002..rtf]

Supplementary Table 3. Joinpoint analysis for oral cavity and pharynx, esophagus, stomach, colorectum, liver, pancreas, larynx, lung, breast, uterus, ovary, bladder, kidney, non-Hodgkin lymphoma, multiple myeloma, leukemia and all cancers in women at age groups 65-69, 70-74, 75-79, 80-84, 85-89, 90-94, 95+ years, in selected worldwide countries.

Cancer site	Country	Year	Age	APC 1	Age	APC 2	AAPC	
ORAL CAVITY AND PHARYNX	USA	2000-2014	(65-69)--(95+)	31.3*			31.3*	
	Japan	2000-2014	(65-69)--(95+)	51.8*			51.8*	
	Australia	2000-2014	(65-69)--(95+)	35.1*			35.1*	
	UK	2000-2014	(65-69)--(95+)	28.1*			28.1*	
	Germany	2000-2014	(65-69)--(95+)	21.6*			21.6*	
	France	2000-2014	(65-69)--(75-79)	10.2	(75-79)--(95+)	39.5*	29*	
	Italy	2000-2014	(65-69)--(95+)	37.7*			37.7*	
	Poland	2000-2014	(65-69)--(95+)	30.3*			30.3*	
ESOPHAGUS	USA	2000-2014	(65-69)--(95+)	24.1*			24.1*	
	Japan	2000-2014	(65-69)--(95+)	36.4*			36.4*	
	Australia	2000-2014	(65-69)--(85-89)	56.2*	(85-89)--(95+)	-3.3	33.1*	
	UK	2000-2014	(65-69)--(80-84)	55.6*	(80-84)--(95+)	9	30.2*	
	Germany	2000-2014	(65-69)--(95+)	25.1*			25.1*	
	France	2000-2014	(65-69)--(95+)	28.1*			28.1*	
	Italy	2000-2014	(65-69)--(85-89)	45.3*	(85-89)--(95+)	-1.9	27.5*	
	Poland	2000-2014	(65-69)--(95+)	26*			26*	
STOMACH	USA	2000-2014	(65-69)--(85-89)	43.3*	(85-89)--(95+)	15.2	33.3*	
	Japan	2000-2014	(65-69)--(95+)	49.9*			49.9*	
	Australia	2000-2014	(65-69)--(85-89)	51.7*	(85-89)--(95+)	-5.5	29.6*	
	UK	2000-2014	(65-69)--(80-84)	64.8*	(80-84)--(95+)	11	35.2*	
	Germany	2000-2014	(65-69)--(85-89)	55*	(85-89)--(95+)	12.1*	39.1*	
	France	2000-2014	(65-69)--(95+)	48.7*			48.7*	
	Italy	2000-2014	(65-69)--(85-89)	55.2*	(85-89)--(95+)	5.8	36.6*	
	Poland	2000-2014	(65-69)--(80-84)	42.8*	(80-84)--(95+)	-0.6	19.2*	
COLORECTUM	USA	2000-2014	(65-69)--(85-89)	45.9*	(85-89)--(95+)	27.9*	39.6*	
	Japan	2000-2014	(65-69)--(95+)	48.1*			48.1*	
	Australia	2000-2014	(65-69)--(85-89)	49.5*	(85-89)--(95+)	10.9*	35.3*	
	UK	2000-2014	(65-69)--(85-89)	51.9*	(85-89)--(95+)	5	34.3*	
	Germany	2000-2014	(65-69)--(85-89)	58.8*	(85-89)--(95+)	13.3	41.9*	
	France	2000-2014	(65-69)--(95+)	51.5*			51.5*	
	Italy	2000-2014	(65-69)--(85-89)	53.8*	(85-89)--(95+)	9	37.1*	
	Poland	2000-2014	(65-69)--(80-84)	45.4*	(80-84)--(95+)	6.6	24.5*	
LIVER	USA	2000-2014	(65-69)--(80-84)	28.6*	(80-84)--(95+)	-5.4	10.3*	
	Japan	2000-2014	(65-69)--(75-79)	64.8*	(75-79)--(95+)	8.4	24.6*	
	Australia	2000-2014	(65-69)--(80-84)	38.4*	(80-84)--(95+)	-3.5	15.6*	
	UK	2000-2014	(65-69)--(85-89)	34*	(85-89)--(95+)	-21.3	12.2	
	Germany	2000-2014	(65-69)--(80-84)	41.5*	(80-84)--(95+)	-7.2	14.6*	
	France	2000-2014	(65-69)--(80-84)	37.6*	(80-84)--(95+)	-5.3	14.2*	
	Italy	2000-2014	(65-69)--(80-84)	47.2*	(80-84)--(95+)	-20.1	8.5	
	Poland	2000-2014	(65-69)--(85-89)	34.1*	(85-89)--(95+)	-39.3	2.9	
PANCREAS	USA	2000-2014	(65-69)--(80-84)	38*	(80-84)--(95+)	9.9	23.1*	
	Japan	2000-2014	(65-69)--(85-89)	42.6*	(85-89)--(95+)	0.3	26.8*	
	Australia	2000-2014	(65-69)--(80-84)	47.6*	(80-84)--(95+)	5.4	24.8*	
	UK	2000-2014	(65-69)--(80-84)	40.2*	(80-84)--(95+)	3	20.2*	
	Germany	2000-2014	(65-69)--(85-89)	37.2*	(85-89)--(95+)	-14.6	17.2*	
	France	2000-2014	(65-69)--(80-84)	41.5*	(80-84)--(95+)	15.7*	28*	
	Italy	2000-2014	(65-69)--(85-89)	38.9*	(85-89)--(95+)	-14.2	18.3*	
	Poland	2000-2014	(65-69)--(80-84)	31.1*	(80-84)--(95+)	-3.4	12.5*	
LARYNX	USA	2000-2014	(65-69)--(75-79)	22*	(75-79)--(95+)	-8.1*	1	
	Japan	2000-2014	(65-69)--(95+)	44.5*			44.5*	
	Australia	2000-2014	(65-69)--(95+)	10.6			10.6	
	UK	2000-2014	(65-69)--(95+)	17.7*			17.7*	
	Germany	2000-2014	(65-69)--(95+)	12.8*			12.8*	
	France	2000-2014	(65-69)--(95+)	16.2*			16.2*	
	Italy	2000-2014	(65-69)--(95+)	27.4*			27.4*	
	Poland	2000-2014	(65-69)--(95+)	-5.1			-5.1	
LUNG	USA	2000-2014	(65-69)--(75-79)	33.8*	(75-79)--(95+)	-6.7	5.2*	
	Japan	2000-2014	(65-69)--(85-89)	47.1*	(85-89)--(95+)	9.2	33.2*	
	Australia	2000-2014	(65-69)--(80-84)	28.4*	(80-84)--(95+)	-17.1	3.2	
	UK	2000-2014	(65-69)--(80-84)	29.2*	(80-84)--(95+)	-18.5	2.6	
	Germany	2000-2014	(65-69)--(80-84)	17.2*	(80-84)--(95+)	-10.2	2.5	
	France	2000-2014	(65-69)--(80-84)	18.1*	(80-84)--(95+)	-3.1	6.9*	
	Italy	2000-2014	(65-69)--(80-84)	30.1*	(80-84)--(95+)	-6	10.6*	
	Poland	2000-2014	(65-69)--(80-84)	5.2	(80-84)--(95+)	-12.8	-4.2	
BREAST	USA	2000-2014	(65-69)--(80-84)	23*	(80-84)--(95+)	26.6*	24.8*	
	Japan	2000-2014	(65-69)--(75-79)	-1.8	(75-79)--(95+)	22.1*	13.5*	
	Australia	2000-2014	(65-69)--(95+)	25.5*			25.5*	
	UK	2000-2014	(65-69)--(95+)	32.4*			32.4*	
	Germany	2000-2014	(65-69)--(95+)	25.3*			25.3*	
	France	2000-2014	(65-69)--(80-84)	22.2*	(80-84)--(95+)	34.4*	28.1*	
	Italy	2000-2014	(65-69)--(95+)	27.5*			27.5*	
	Poland	2000-2014	(65-69)--(95+)	18.5*			18.5*	
UTERUS (CERVIX AND CORPUS)	USA	2000-2014	(65-69)--(95+)	13.6*			13.6*	
	Japan	2000-2014	(65-69)--(95+)	23.7*			23.7*	
	Australia	2000-2014	(65-69)--(85-89)	26.4*	(85-89)--(95+)	-1.6	16.3*	
	UK	2000-2014	(65-69)--(85-89)	24.2*	(85-89)--(95+)	-8.7	12.1*	
	Germany	2000-2014	(65-69)--(85-89)	30.8*	(85-89)--(95+)	1.8	20.3*	
	France	2000-2014	(65-69)--(80-84)	29.7*	(80-84)--(95+)	15.1	22.2*	
	Italy	2000-2014	(65-69)--(85-89)	27.1*	(85-89)--(95+)	-7.9	14.2*	
	Poland	2000-2014	(65-69)--(85-89)	11.7*	(85-89)--(95+)	-29.1*	-4	
OVARY	USA	2000-2014	(65-69)--(80-84)	24.4*	(80-84)--(95+)	-3.6	9.5*	
	Japan	2000-2014	(65-69)--(95+)	14.4*			14.4*	
	Australia	2000-2014	(65-69)--(85-89)	22.8*	(85-89)--(95+)	-21.4	5.9	
	UK	2000-2014	(65-69)--(85-89)	14.3*	(85-89)--(95+)	-22.7	0.3	
	Germany	2000-2014	(65-69)--(80-84)	26.2*	(80-84)--(95+)	-7	8.3*	
	France	2000-2014	(65-69)--(80-84)	21*	(80-84)--(95+)	1.8	11*	
	Italy	2000-2014	(65-69)--(85-89)	17.9*	(85-89)--(95+)	-26.1	0.9	
	Poland	2000-2014	(65-69)--(80-84)	6.5*	(80-84)--(95+)	-17.5	-6.3*	
BLADDER	USA	2000-2014	(65-69)--(85-89)	59.8*	(85-89)--(95+)	28.3	48.5*	
	Japan	2000-2014	(65-69)--(85-89)	90.5*	(85-89)--(95+)	29.1*	67.3*	
	Australia	2000-2014	(65-69)--(85-89)	71.9*	(85-89)--(95+)	14	49.9*	
	UK	2000-2014	(65-69)--(85-89)	65.6*	(85-89)--(95+)	9.1	44.1*	
	Germany	2000-2014	(65-69)--(85-89)	75.9*	(85-89)--(95+)	15*	52.6*	
	France	2000-2014	(65-69)--(85-89)	72.1*	(85-89)--(95+)	36.8*	59.4*	
	Italy	2000-2014	(65-69)--(85-89)	73.3*	(85-89)--(95+)	15.1	51.2*	
	Poland	2000-2014	(65-69)--(80-84)	54.7*	(80-84)--(95+)	17.6*	34.9*	
KIDNEY AND OTHER URINARY SITES	USA	2000-2014	(65-69)--(80-84)	37.4*	(80-84)--(95+)	14	25.2*	
	Japan	2000-2014	(65-69)--(85-89)	55.5*	(85-89)--(95+)	6.4	37.1*	
	Australia	2000-2014	(65-69)--(85-89)	43.5*	(85-89)--(95+)	-11.1	22.3*	
	UK	2000-2014	(65-69)--(85-89)	34.3*	(85-89)--(95+)	-14.3	15.6*	
	Germany	2000-2014	(65-69)--(85-89)	41.3*	(85-89)--(95+)	-15.4	19*	
	France	2000-2014	(65-69)--(85-89)	43.2*	(85-89)--(95+)	8.2	30.5*	
	Italy	2000-2014	(65-69)--(85-89)	44.8*	(85-89)--(95+)	-20.2	18.8*	
	Poland	2000-2014	(65-69)--(80-84)	31.1*	(80-84)--(95+)	-14.7	5.8	
NON-HODGKIN LYMPHOMA	USA	2000-2014	(65-69)--(80-84)	56.1*	(80-84)--(95+)	10.9	31.6*	
	Japan	2000-2014	(65-69)--(85-89)	54.5*	(85-89)--(95+)	-12.5	27.8*	
	Australia	2000-2014	(65-69)--(85-89)	52.1*	(85-89)--(95+)	-17.6	24*	
	UK	2000-2014	(65-69)--(80-84)	47.2*	(80-84)--(95+)	3.6	23.5*	
	Germany	2000-2014	(65-69)--(80-84)	56*	(80-84)--(95+)	-1.4	24*	
	France	2000-2014	(65-69)--(80-84)	56*	(80-84)--(95+)	15.1	34*	
	Italy	2000-2014	(65-69)--(80-84)	55.4*	(80-84)--(95+)	-6.3	20.6*	
	Poland	2000-2014	(65-69)--(80-84)	28.6*	(80-84)--(95+)	-19.5	1.8	
MULTIPLE MYELOMA	USA	2000-2014	(65-69)--(80-84)	43.4*	(80-84)--(95+)	-0.7	19.4*	
	Japan	2000-2014	(65-69)--(80-84)	52.7*	(80-84)--(95+)	-2.3	22.1*	
	Australia	2000-2014	(65-69)--(80-84)	55*	(80-84)--(95+)	0.7	24.9*	
	UK	2000-2014	(65-69)--(85-89)	40.5*	(85-89)--(95+)	-16.5	18.1*	
	Germany	2000-2014	(65-69)--(80-84)	42.7*	(80-84)--(95+)	-14.6	10.4*	
	France	2000-2014	(65-69)--(85-89)	43.9*	(85-89)--(95+)	-4.2	25.6*	
	Italy	2000-2014	(65-69)--(80-84)	50.7*	(80-84)--(95+)	-6.4	18.8*	
	Poland	2000-2014	(65-69)--(75-79)	44.2	(75-79)--(95+)	-13.5	2.6	
LEUKEMIA	USA	2000-2014	(65-69)--(80-84)	51*	(80-84)--(95+)	24.1*	36.9*	
	Japan	2000-2014	(65-69)--(85-89)	33*	(85-89)--(95+)	-10.8	16.4*	
	Australia	2000-2014	(65-69)--(85-89)	49.7*	(85-89)--(95+)	6	33.4*	
	UK	2000-2014	(65-69)--(85-89)	46.5*	(85-89)--(95+)	8.7	32.6*	
	Germany	2000-2014	(65-69)--(85-89)	47.4*	(85-89)--(95+)	-9.8	25.2*	
	France	2000-2014	(65-69)--(85-89)	51.6*	(85-89)--(95+)	21.5	40.8*	
	Italy	2000-2014	(65-69)--(85-89)	46.1*	(85-89)--(95+)	-5	26.5*	
	Poland	2000-2014	(65-69)--(80-84)	37.4*	(80-84)--(95+)	1.8	18.3*	
ALL CANCERS	USA	2000-2014	(65-69)--(75-79)	37.8*	(75-79)--(95+)	17.7*	24.1*	
	Japan	2000-2014	(65-69)--(85-89)	46.8*	(85-89)--(95+)	16.9*	36.1*	
	Australia	2000-2014	(65-69)--(85-89)	38.4*	(85-89)--(95+)	1.1	24.6*	
	UK	2000-2014	(65-69)--(85-89)	35.3*	(85-89)--(95+)	-1.6	21.7*	
	Germany	2000-2014	(65-69)--(85-89)	38.5*	(85-89)--(95+)	4	25.9*	
	France	2000-2014	(65-69)--(85-89)	38*	(85-89)--(95+)	26.9*	34.2*	
	Italy	2000-2014	(65-69)--(85-89)	40.4*	(85-89)--(95+)	0.3	25.5*	
	Poland	2000-2014	(65-69)--(85-89)	25.5*	(85-89)--(95+)	-12.8	11.2*	


* significantly different from 0 (p < 0.05)
APC, estimated annual percent change
AAPC, estimated average annual percent change
