# Supplementary material for: Cancer mortality in the oldest old: a global overview
Source: Aging (Albany NY). 2020 Sep 3;12(17):16744–58. doi: 10.18632/aging.103503 (PMC7521488; doi:10.18632/aging.103503)
Supplement: Supplementary Table 3 [file aging-12-103503-s003..rtf]

Supplementary Table 2. Joinpoint analysis for oral cavity and pharynx, esophagus, stomach, colorectum, liver, pancreas, larynx, lung, prostate, bladder, kidney, non-Hodgkin lymphoma, multiple myeloma, leukemia and all cancers in men at age groups 65-69, 70-74, 75-79, 80-84, 85-89, 90-94, 95+ years, in selected worldwide countries.

Cancer site	Country	Year	Age	APC 1	Age	APC 2	AAPC	
ORAL CAVITY AND PHARYNX	USA	2000-2014	(65-69)--(80-84)	14.7*	(80-84)--(95+)	18.7*	16.7*	
	Japan	2000-2014	(65-69)--(85-89)	23.5*	(85-89)--(95+)	0.8	15.4*	
	Australia	2000-2014	(65-69)--(95+)	14.4*			14.4*	
	UK	2000-2014	(65-69)--(95+)	11.2*			11.2*	
	Germany	2000-2014	(65-69)--(80-84)	-6.8	(80-84)--(95+)	14.3	3.2	
	France	2000-2014	(65-69)--(80-84)	0	(80-84)--(95+)	8.3	4.1*	
	Italy	2000-2014	(65-69)--(95+)	13.9*			13.9*	
	Poland	2000-2014	(65-69)--(95+)	-2.2			-2.2	
ESOPHAGUS	USA	2000-2014	(65-69)--(80-84)	20.7*	(80-84)--(95+)	-2	8.8*	
	Japan	2000-2014	(65-69)--(95+)	12.9*			12.9*	
	Australia	2000-2014	(65-69)--(85-89)	28.4*	(85-89)--(95+)	-21.2	9.1*	
	UK	2000-2014	(65-69)--(85-89)	29.6*	(85-89)--(95+)	-18	11.3*	
	Germany	2000-2014	(65-69)--(85-89)	8.2*	(85-89)--(95+)	-16.8	-0.9	
	France	2000-2014	(65-69)--(75-79)	18.8*	(75-79)--(95+)	5.2	9.6*	
	Italy	2000-2014	(65-69)--(80-84)	24.9*	(80-84)--(95+)	-1.5	10.9*	
	Poland	2000-2014	(65-69)--(95+)	2			2	
STOMACH	USA	2000-2014	(65-69)--(95+)	33.6*			33.6*	
	Japan	2000-2014	(65-69)--(85-89)	42.5*	(85-89)--(95+)	11	31.1*	
	Australia	2000-2014	(65-69)--(85-89)	42.1*	(85-89)--(95+)	-10.5	21.8*	
	UK	2000-2014	(65-69)--(80-84)	55.4*	(80-84)--(95+)	5.4	28*	
	Germany	2000-2014	(65-69)--(85-89)	44.9*	(85-89)--(95+)	-5.5	25.6*	
	France	2000-2014	(65-69)--(85-89)	39.3*	(85-89)--(95+)	9.5	28.5*	
	Italy	2000-2014	(65-69)--(80-84)	48*	(80-84)--(95+)	8.6	26.7*	
	Poland	2000-2014	(65-69)--(80-84)	30.2*	(80-84)--(95+)	-18.1	3.2	
COLORECTUM	USA	2000-2014	(65-69)--(80-84)	40.3*	(80-84)--(95+)	29.9*	35*	
	Japan	2000-2014	(65-69)--(85-89)	39.2*	(85-89)--(95+)	20.8	32.7*	
	Australia	2000-2014	(65-69)--(80-84)	44.6*	(80-84)--(95+)	18	30.6*	
	UK	2000-2014	(65-69)--(85-89)	44.8*	(85-89)--(95+)	-1.5	27.3*	
	Germany	2000-2014	(65-69)--(85-89)	42.8*	(85-89)--(95+)	-1.7	26.1*	
	France	2000-2014	(65-69)--(80-84)	49.9*	(80-84)--(95+)	29.7*	39.4*	
	Italy	2000-2014	(65-69)--(80-84)	53.1*	(80-84)--(95+)	13	31.5*	
	Poland	2000-2014	(65-69)--(80-84)	36.9*	(80-84)--(95+)	-13.6	8.8	
LIVER	USA	2000-2014	(65-69)--(80-84)	19.3*	(80-84)--(95+)	-7.8*	4.9*	
	Japan	2000-2014	(65-69)--(75-79)	36.1*	(75-79)--(95+)	2.2	12.5*	
	Australia	2000-2014	(65-69)--(80-84)	31.4*	(80-84)--(95+)	-13.3	6.7*	
	UK	2000-2014	(65-69)--(80-84)	33.2*	(80-84)--(95+)	-5.9	12*	
	Germany	2000-2014	(65-69)--(95+)	15.7*			15.7*	
	France	2000-2014	(65-69)--(75-79)	26.6*	(75-79)--(95+)	-9.1	1.6	
	Italy	2000-2014	(65-69)--(80-84)	29*	(80-84)--(95+)	-25.3	-1.8	
	Poland	2000-2014	(65-69)--(75-79)	27.5	(75-79)--(95+)	-5.3	4.6	
PANCREAS	USA	2000-2014	(65-69)--(80-84)	31.1*	(80-84)--(95+)	7.8	18.9*	
	Japan	2000-2014	(65-69)--(80-84)	35.3*	(80-84)--(95+)	9.1*	21.5*	
	Australia	2000-2014	(65-69)--(80-84)	36.6*	(80-84)--(95+)	4.5	19.5*	
	UK	2000-2014	(65-69)--(80-84)	34.1*	(80-84)--(95+)	2.6	17.3*	
	Germany	2000-2014	(65-69)--(80-84)	31*	(80-84)--(95+)	-0.6	14.1*	
	France	2000-2014	(65-69)--(75-79)	29.9*	(75-79)--(95+)	13.3*	18.6*	
	Italy	2000-2014	(65-69)--(80-84)	29.6*	(80-84)--(95+)	-4.8	11*	
	Poland	2000-2014	(65-69)--(80-84)	17.7*	(80-84)--(95+)	-17.5	-1.5	
LARYNX	USA	2000-2014	(65-69)--(75-79)	20.9*	(75-79)--(95+)	11*	14.2*	
	Japan	2000-2014	(65-69)--(85-89)	45*	(85-89)--(95+)	-0.6	27.9*	
	Australia	2000-2014	(65-69)--(95+)	21.1*			21.1*	
	UK	2000-2014	(65-69)--(95+)	23.4*			23.4*	
	Germany	2000-2014	(65-69)--(95+)	10.7*			10.7*	
	France	2000-2014	(65-69)--(95+)	10.3*			10.3*	
	Italy	2000-2014	(65-69)--(85-89)	30.8*	(85-89)--(95+)	-16.2	12.7	
	Poland	2000-2014	(65-69)--(80-84)	0.2	(80-84)--(95+)	-32.6	-17.8*	
LUNG	USA	2000-2014	(65-69)--(75-79)	37.5*	(75-79)--(95+)	2.1	12.7*	
	Japan	2000-2014	(65-69)--(80-84)	56.2*	(80-84)--(95+)	6.2	28.8*	
	Australia	2000-2014	(65-69)--(80-84)	38.5*	(80-84)--(95+)	-9.9	11.7	
	UK	2000-2014	(65-69)--(80-84)	37.3*	(80-84)--(95+)	-5.8	13.7*	
	Germany	2000-2014	(65-69)--(80-84)	24*	(80-84)--(95+)	-19.2	0.1	
	France	2000-2014	(65-69)--(75-79)	21.5*	(75-79)--(95+)	-2.1	5.2*	
	Italy	2000-2014	(65-69)--(80-84)	34.9*	(80-84)--(95+)	-19.9	3.9	
	Poland	2000-2014	(65-69)--(75-79)	18.8*	(75-79)--(95+)	-22.2*	-10.4*	
PROSTATE	USA	2000-2014	(65-69)--(85-89)	80.2*	(85-89)--(95+)	44.3*	67.3*	
	Japan	2000-2014	(65-69)--(80-84)	102.1*	(80-84)--(95+)	51.1*	74.7*	
	Australia	2000-2014	(65-69)--(80-84)	101.1*	(80-84)--(95+)	39.6	67.5*	
	UK	2000-2014	(65-69)--(85-89)	83.2*	(85-89)--(95+)	19.8	59*	
	Germany	2000-2014	(65-69)--(85-89)	80.1*	(85-89)--(95+)	9.7	52.7*	
	France	2000-2014	(65-69)--(85-89)	86.6*	(85-89)--(95+)	45.6	71.8*	
	Italy	2000-2014	(65-69)--(85-89)	91.7*	(85-89)--(95+)	14.3*	61.3*	
	Poland	2000-2014	(65-69)--(80-84)	74.7*	(80-84)--(95+)	6.8	36.6*	
BLADDER	USA	2000-2014	(65-69)--(85-89)	64.7*	(85-89)--(95+)	35*	54.1*	
	Japan	2000-2014	(65-69)--(85-89)	82.5*	(85-89)--(95+)	36.7*	65.8*	
	Australia	2000-2014	(65-69)--(85-89)	74.9*	(85-89)--(95+)	15.9	52.5*	
	UK	2000-2014	(65-69)--(85-89)	69.3*	(85-89)--(95+)	17.3	49.8*	
	Germany	2000-2014	(65-69)--(85-89)	75.5*	(85-89)--(95+)	10	50.2*	
	France	2000-2014	(65-69)--(85-89)	54.1*	(85-89)--(95+)	29.9	45.6*	
	Italy	2000-2014	(65-69)--(85-89)	65.6*	(85-89)--(95+)	-5.5	37.4*	
	Poland	2000-2014	(65-69)--(80-84)	45.6*	(80-84)--(95+)	-1.6	19.6*	
KIDNEY AND OTHER URINARY SITES	USA	2000-2014	(65-69)--(85-89)	29.1*	(85-89)--(95+)	9.6	22.3*	
	Japan	2000-2014	(65-69)--(80-84)	50.1*	(80-84)--(95+)	19.3	33.8*	
	Australia	2000-2014	(65-69)--(95+)	37.7*			37.7*	
	UK	2000-2014	(65-69)--(85-89)	34.9*	(85-89)--(95+)	-7.3	19.1*	
	Germany	2000-2014	(65-69)--(85-89)	41*	(85-89)--(95+)	-19.5	17	
	France	2000-2014	(65-69)--(95+)	34*			34*	
	Italy	2000-2014	(65-69)--(80-84)	45*	(80-84)--(95+)	1.4	21.3*	
	Poland	2000-2014	(65-69)--(95+)	11.7*			11.7*	
NON-HODGKIN LYMPHOMA	USA	2000-2014	(65-69)--(80-84)	52.1*	(80-84)--(95+)	16.1	32.9*	
	Japan	2000-2014	(65-69)--(80-84)	59.1*	(80-84)--(95+)	15.2	35.4*	
	Australia	2000-2014	(65-69)--(80-84)	55.4*	(80-84)--(95+)	12	31.9*	
	UK	2000-2014	(65-69)--(80-84)	47.2*	(80-84)--(95+)	7.4	25.7*	
	Germany	2000-2014	(65-69)--(80-84)	52.4*	(80-84)--(95+)	1.4	24.3*	
	France	2000-2014	(65-69)--(80-84)	50.9*	(80-84)--(95+)	18.3	33.6*	
	Italy	2000-2014	(65-69)--(80-84)	48.7*	(80-84)--(95+)	-4.6	19.1*	
	Poland	2000-2014	(65-69)--(75-79)	37.8*	(75-79)--(95+)	-9.5	4.1	
MULTIPLE MYELOMA	USA	2000-2014	(65-69)--(80-84)	45.9*	(80-84)--(95+)	7.5	25.2*	
	Japan	2000-2014	(65-69)--(80-84)	55.5*	(80-84)--(95+)	11	31.4*	
	Australia	2000-2014	(65-69)--(85-89)	47.6*	(85-89)--(95+)	-19	20.9*	
	UK	2000-2014	(65-69)--(80-84)	53.7*	(80-84)--(95+)	9.7	29.8*	
	Germany	2000-2014	(65-69)--(80-84)	43.6*	(80-84)--(95+)	-6.3	16*	
	France	2000-2014	(65-69)--(80-84)	55.9*	(80-84)--(95+)	21.8	37.8*	
	Italy	2000-2014	(65-69)--(80-84)	53.5*	(80-84)--(95+)	4	26.4*	
	Poland	2000-2014	(65-69)--(75-79)	44.6	(75-79)--(95+)	-7.8	7.1	
LEUKEMIA	USA	2000-2014	(65-69)--(80-84)	53.4*	(80-84)--(95+)	20*	35.7*	
	Japan	2000-2014	(65-69)--(80-84)	41.6*	(80-84)--(95+)	2.6	20.6*	
	Australia	2000-2014	(65-69)--(80-84)	60.4*	(80-84)--(95+)	14.2	35.4*	
	UK	2000-2014	(65-69)--(80-84)	52.3*	(80-84)--(95+)	15.8	32.8*	
	Germany	2000-2014	(65-69)--(80-84)	54*	(80-84)--(95+)	9.5	29.8*	
	France	2000-2014	(65-69)--(80-84)	57.7*	(80-84)--(95+)	27.6*	41.9*	
	Italy	2000-2014	(65-69)--(80-84)	56.3*	(80-84)--(95+)	8.8	30.4*	
	Poland	2000-2014	(65-69)--(80-84)	39.4*	(80-84)--(95+)	-7*	13.9*	
ALL CANCERS	USA	2000-2014	(65-69)--(80-84)	39.2*	(80-84)--(95+)	21.4*	30*	
	Japan	2000-2014	(65-69)--(80-84)	47.4*	(80-84)--(95+)	19.9*	32.9*	
	Australia	2000-2014	(65-69)--(80-84)	51.5*	(80-84)--(95+)	17.8	33.6*	
	UK	2000-2014	(65-69)--(85-89)	43.7*	(85-89)--(95+)	-0.8	27*	
	Germany	2000-2014	(65-69)--(85-89)	37.8*	(85-89)--(95+)	-5.7	21.4*	
	France	2000-2014	(65-69)--(95+)	33.3*			33.3*	
	Italy	2000-2014	(65-69)--(80-84)	47*	(80-84)--(95+)	8.8	26.5*	
	Poland	2000-2014	(65-69)--(80-84)	25.8*	(80-84)--(95+)	-12.6	4.9	


* significantly different from 0 (p < 0.05)
APC, estimated annual percent change
AAPC, estimated average annual percent change
